# Vascular plant community composition from the *campos rupestres* of the Itacolomi State Park, Brazil

**DOI:** 10.3897/BDJ.3.e4507

**Published:** 2015-02-27

**Authors:** Markus Gastauer, Werner Leyh, Angela S. Miazaki, João A.A. Meira-Neto

**Affiliations:** ‡ Federal University of Viçosa, Frutal, Brazil; § Centro de Ciências Ambientais Floresta-Escola, Frutal, Brazil; | Federal University of Viçosa, Viçosa, Brazil

**Keywords:** Endemic species, endangered ecosystems, anthropogenic impacts, Braun-Blanquet, soil properties, Espinhaço Mountain Range, Wilmanns cover-abundance scale, species cardinality

## Abstract

*Campos rupestres* are rare and endangered ecosystems that accommodate a species-rich flora with a high degree of endemism. Here, we make available a dataset from phytosociological surveys carried out in the Itacolomi State Park, Minas Gerais, southeastern Brazil. All species in a total of 30 plots of 10 x 10 m from two study sites were sampled. Their cardinality, a combination of cover and abundance, was estimated. Altogether, we registered occurrences from 161 different taxa from 114 genera and 47 families. The families with the most species were Poaceae and Asteraceae, followed by Cyperaceae. Abiotic descriptions, including soil properties such as type, acidity, nutrient or aluminum availability, cation exchange capacity, and saturation of bases, as well as the percentage of rocky outcrops and the mean inclination for each plot, are given. This dataset provides unique insights into the *campo rupestre* vegetation, its specific environment and the distribution of its diversity.

## Introduction

*Campos rupestres* (literally: rock fields) are rare and endangered ecosystems composed of different physiognomies (Caiafa and Silva 2005) on quartzite material or sandstone, which displace forest formations in high altitudes (900 m above sea level) in southeastern Brazil (Magalhães 1966). These rock fields are considered species-rich, diverse ecosystems that contain many endemics (Giulietti and Pirani 1988, Pirani et al. 1994, Stannard 1995, Romero and Nakajima 1999, Conceição et al. 2007, Jacobi et al. 2007, Menini Neto et al. 2007, Messias et al. 2011​). Nevertheless, the intensification of grazing, uncontrollable fires of anthropogenic origin, continued urbanization, dispersion of invasive plant species, collecting of endangered (medical) plants, mining activities and nutrient inputs from industry and traffic threaten the native flora and fauna (Pirani et al. 2003, Gastauer et al. 2012).

The Itacolomi State Park (ISP) is located in the Brazilian municipalities of Mariana and Ouro Preto, Minas Gerais state, south of the Espinhaço Mountain Range (Drummond 2005). Due to the characteristic mosaic of different physiognomies of *campo rupestre* vegetation and the seasonal semideciduous Atlantic forest (Peron 1989, Gastauer and Meira Neto 2013), the park contains a diverse flora with many endemics (Batista et al. 2004, Dutra et al. 2008, Dutra et al. 2009).

Although *campos rupestres* contain a high degree of endemic species, little data are available for this endangered ecosystem (Gastauer and Meira Neto 2013). The aim of this data paper is the distribution of a data set containing species lists from two *campo rupestre* communities from the ISP along with a list of soil parameters to increase knowledge of the actual distribution of *campo rupestre* species.

## Project description

### Title

Species richness, diversity and community composition of *campo rupestre* vegetation in the Itacolomi State Park

### Study area description

**Figure 1.**
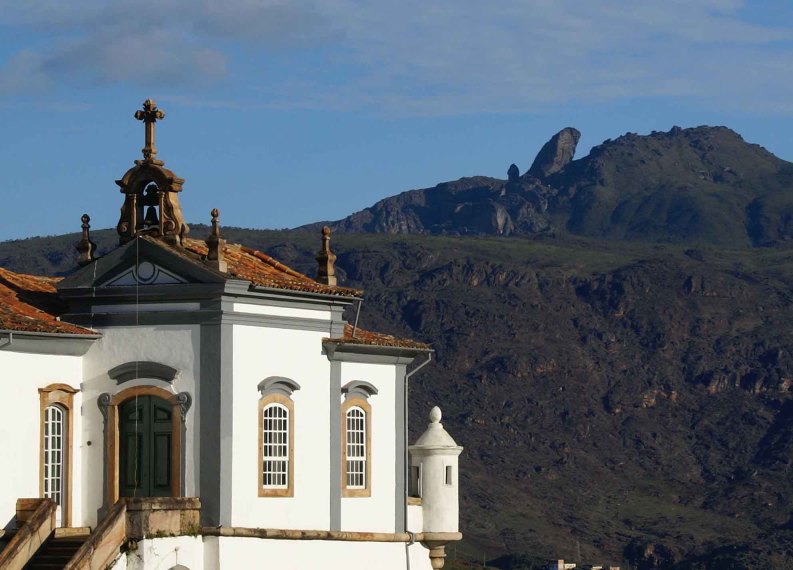
The Itacolomi peak, photographed from the historical center of Ouro Preto, Brazil.

This study was carried out in the ISP. Founded in 1967, the ISP covers 7543 ha in the southern part of the Espinaço Mountain Range. The park's name is derived from its highest peak, the *Pico de Itacolomi* (1722 m above sea level), which means “little stone girl” in the Tupi Indian language. This is a reference to the characteristic rocks forming the peak, which are considered to be a mother and daughter by native people (Fig. 1).

The vegetation of the park is formed by a mosaic of seasonal semideciduous Atlantic forest and *campos rupestres* (Fujaco et al. 2010). In a recent census, which included this survey, 520 species had already been recorded for the ISP, but the magnitude of the total species richness of vascular plants is estimated to be between 880 and 1340 species (Gastauer and Meira Neto 2013). *Habernaria itaculumia* (Orchidaceae, Batista et al. 2004) and *Chamaecrista dentata* (Fabaceae, Dutra et al. 2008) are species endemic to the park.

Soils are of quartzitic origin, and rocky outcrops are distributed over both study sites. The climate of the park is of type Cwb, according to the Köppen classification (Peel et al. 2007). The climate is mesothermic, with mild, rainy summers and dry winters. The average temperature ranges between 17 and 18.5° C, and the annual precipitation reaches 1450 to 1800 mm (Werneck et al. 2000).

**Figure 2.**
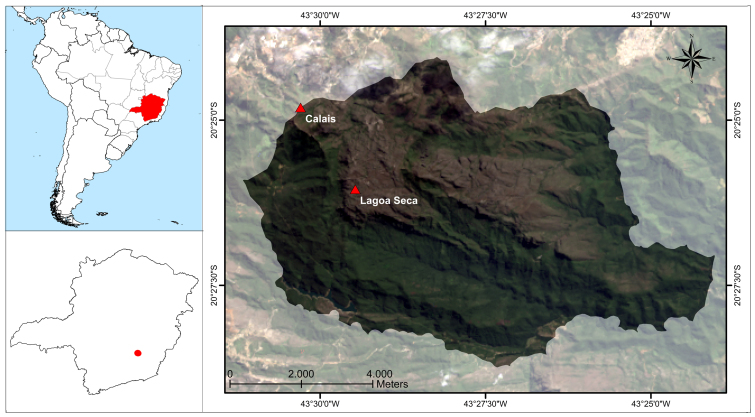
Geographical position of the study sites within the ISP.

Two study sites were selected. The first study site, Lagoa Seca (Dry Pond in English), is situated near a periodically inundated area at the coordinates 20°25.96'S and 43°29.47'W, 1600 m above sea level in the center of the ISP (Fig. 2). The second study site, Calais, is situated near the boundary of the ISP at 20°24.61'S and 43°30.13'W, at an altitude of 1270 m. Both areas show a homogeneous, small-scale mosaic of gramineous vegetation, small shrubs and quartzite outcrops. Although the Lagoa Seca study site is well protected within the park, it burned in 2007 (Fundação Biodiversitas, personal communication), and the Calais area is impacted by invading cattle and fires that frequently become out of control in neighboring areas. Furthermore, settlement activities disturb the area with waste deposits from urban households and construction activities.

### Funding

JAAMN received a CNPq scholarship.

## Sampling methods

### Sampling description

At each study site, 15 plots of 10 x 10 m, arranged in 3 transects, were installed (Newton 2007). In each plot, the complete vegetation cover, its mean inclination and aspect, i.e., the compass direction that the slopes face, were estimated.

The cardinality, a combination of abundance and vegetation cover, of each species within each plot was estimated using the Wilmanns scale (Reichelt and Wilmanns 1973). This scale is easily converted to the more common, internationally accepted Braun-Blanquet system (Mueller-Dombois and Ellenberg 1974, Table 1).

Unknown species were collected and identified with the help of specialists, and a specimen from each was deposited in the OUPR herbarium from the Federal University of Ouro Preto (UFOP).

Soil samples were collected in each plot. From five equally distributed points in each plot, the upper 20 cm of the soil was removed using a hoe after the organic layers had been removed. The five samples of each plot were mixed, and then 500 g was weighed, stored in a plastic bag and transported to the lab. Immediately after arrival at the lab, the soil samples were air-dried.

The following parameters were analyzed in the laboratories of the Soil Department of the Federal University of Viçosa: soil texture (determination of the relative amounts of course and fine sand, silt and clay, separated by sieving); soil acidity (pH, extraction with water); the concentrations of phosphorus (P), potassium (K, both Mehlich 1 extraction), calcium (Ca), magnesium (Mg), and aluminum (Al, the previous three all extracted with 1 mol/L KCl); interchangeable bases (SB); the effective cation exchange capacity (CTC(t)), as well as the cation exchange capacity at pH 7 (CTC(T)); and the saturation of bases (V), aluminum (M) and remnant phosphorus (P-rem).

## Geographic coverage

### Description

see Fig. 2

### Coordinates

-20.41027 and -20.40948 Latitude; -43.50209 and -43.50138 Longitude.

## Taxonomic coverage

### Description

In Lagoa Seca, we found 76 (morpho-)species from 55 genera and 25 families (Table 2), whereas we found 107 species from 82 genera and 33 families in Calais (Table 3). Due to the lack of appropriate material (e.g., flowers) to provide a definite determination, 15 morphospecies from Lagoa Seca and 13 from Calais were identified to only the genus level, 5 morphospecies from Lagoa Seca and 4 from Calais were identified to only the family level (Tables 2, 3). Altogether, 161 (morpho-) species belonging to 114 genera and 47 families were registered in this study. Most of them were angiosperms (156 species), but six fern and a lycophyte species were recorded as well.

20 (morpho)species occur in both study sites, and these belong to the families Asteraceae (6 species), Poaceae (5), and Cyperaceae (3), with *Mesophaerum homolophylla* (Lamiaceae), *Byrsonima variabilis* (Malpighiaceae), *Cambessedesia hilariana* (Melastomataceae), *Myrcia splendens* (Myrtaceae), *Polygala paniculata* (Polygalaceae) and *Solanum granuloso-leprosum* (Solanaceae) being the sole representatives of their families.

The most dominant families in Lagoa Seca are Asteraceae (with 13 species), Poaceae (11), Cyperaceae (9), Melastomataceae (7) and Orchidaceae (6). Asteraceae and Poaceae, each with 19 species, are the most species-rich families found in the Calais study site, followed by Fabaceae (13), Cyperaceae (9) and Melastomataceae (6). The family Fabaceae, well-represented in Calais, is completely lacking in Lagoa Seca. On the other hand, the family Orchidaceae shows a higher richness in the Lagoa Seca area.

The number of species per plot varies between 16 and 33 in Lagoa Seca and between 21 and 43 in Calais. The number of uniques, i.e., species that occur within one plot only, is high for both study sites. Three species, *Paspalum caryophaeum*, *Schizachyrium sanguine* um and *Rhynchosphora* sp. 1, occur in all 15 plots of Lagoa Seca, but the most dominant species from Calais is *Melinis minutiflora*, occurring in 13 out of 15 plots.

## Temporal coverage

### Notes

Two field campaigns were undertaken to collect soil samples and to survey community composition. The first one took place between the 20^th^ and 22^nd^ of October 2008; the second one was carried out between the 9^th^ and 10^th^ of January 2009. Species not identified during the field work were collected and identified within two or three days after return from the field. Soil samples from both study sites were analyzed in February 2009.

## Usage rights

### Use license

Creative Commons CCZero

### IP rights notes

This dataset can be freely used, provided it is cited.

## Data resources

### Data package title

Composition of campo rupestre communities from the Itacolomi State Park

### Resource link

http://187.32.44.123/ipt/; Calais dataset: http://187.32.44.123/ipt/resource.do?r=camporupestre-15plot-survey-sampling-itacolomi-calais107-checklist; Lagoa Seca dataset: http://187.32.44.123/ipt/resource.do?r=camporupestre-15plot-survey-sampling-itacolomi-lagoa076-checklist

### Alternative identifiers

Calais dataset: http://187.32.44.123/ipt/archive.do?r=camporupestre-15plot-survey-sampling-itacolomi-calais107-checklist, http://www.gbif.org/dataset/7975c522-09d6-47c4-9099-7eab745a71e3; Lagoa Seca dataset: http://187.32.44.123/ipt/archive.do?r=camporupestre-15plot-survey-sampling-itacolomi-lagoa076-checklist, http://www.gbif.org/dataset/8deed7f6-30d2-411d-aee2-e5b5732fb7c3; Download link: http://www.leep.ufv.br/en-US/noticia/dwca-camporupestre-15plot-survey-sampling-itacolomi-for-download

### Number of data sets

2

### Data set 1.

#### Data set name

dwca-camporupestre-15plot-survey-sampling-itacolomi-calais107-checklist.zip

#### Data format

Darwin Core Archive DwC-A

#### Number of columns

29

#### Description

107 species occurrences within and environmental properties of the 15 plots of 10x10m from the Calais study site. Dataset consists of seven independent files (Table 4).

**Data set 1.** 

Column label	Column description
Id	Taxon identifier
taxonID	Taxon identifier
acceptedNameUsageID	Identifier for the name usage
parentNameUsageID	Identifier for the name usage
nameAccordingToID	Identifier for the source in which the specific taxon concept circumscription is defined or implied
scientificName	The full scientific name; when forming only part of an identification, name of lowest level taxonomic rank that was determined
acceptedNameUsage	Full name with authorship information of the sampled taxon
parentNameUsage	Full name of the direct, most proximate higher-rank parent taxon
nameAccordingTo	Reference to the source in which the specific taxon concept circumscription is defined or implied
higherClassification	List of taxa names terminating at the rank immediately superior to the taxon referenced in the taxon record, starting with the highest rank and separating the names for each rank with a semi-colon
kingdom	Full scientific name of the kingdom in which the taxon is classified
class	Full scientific name of the class in which the taxon is classified
order	Full scientific name of the order in which the taxon is classified
family	Full scientific name of the family in which the taxon is classified
genus	Full scientific name of the genus in which the taxon is classified
subgenus	Full scientific name of the subgenus in which the taxon is classified, when available
specificEpithet	Name of the species epithet of the scientificName
infraSpecificEpithet	Name of the lowest or terminal infraspecific epithet of the scientificName
taxonRank	Taxonomic rank of the most specific name in the scientificName
scientificNameAuthorship	Authorship information for the scientificName
nomenclaturalCode	The nomenclatural code under which the scientificName is constructed.
taxonomicStatus	Status of the use of the scientificName as a label for a taxon linked to http://www.tropicos.org/
modified	Date on which the resource was changed
language	The language of the resource.
rights	Information about who can access the resource or an indication of its security status.
rightsHolder	The organization owning and managing rights over the resource.
bibliographicCitation	Bibliography citing this dataset
datasetName	The name identifying the data set from which the record was derived.
references	DOI of bibliography citing this dataset

### Data set 2.

#### Data set name

dwca-camporupestre-15plot-survey-sampling-itacolomi-lagoa076-checklist.zip

#### Data format

Darwin Core Archive DwC-A

#### Number of columns

29

#### Description

76 species occurrences within and environmental properties of the 15 plots of 10x10m from the Lagoa Seca study site. Dataset consists of 6 independent files (Table 4).

**Data set 2.** 

Column label	Column description
Id	Taxon identifier
taxonID	Taxon identifier
acceptedNameUsageID	Identifier for the name usage
parentNameUsageID	Identifier for the name usage
nameAccordingToID	Identifier for the source in which the specific taxon concept circumscription is defined or implied
scientificName	The full scientific name; when forming only part of an identification, name of lowest level taxonomic rank that was determined
acceptedNameUsage	Full name with authorship information of the sampled taxon
parentNameUsage	Full name of the direct, most proximate higher-rank parent taxon
nameAccordingTo	Reference to the source in which the specific taxon concept circumscription is defined or implied
higherClassification	List of taxa names terminating at the rank immediately superior to the taxon referenced in the taxon record, starting with the highest rank and separating the names for each rank with a semi-colon
kingdom	Full scientific name of the kingdom in which the taxon is classified
class	Full scientific name of the class in which the taxon is classified
order	Full scientific name of the order in which the taxon is classified
family	Full scientific name of the family in which the taxon is classified
genus	Full scientific name of the genus in which the taxon is classified
subgenus	Full scientific name of the subgenus in which the taxon is classified, when available
specificEpithet	Name of the species epithet of the scientificName
infraSpecificEpithet	Name of the lowest or terminal infraspecific epithet of the scientificName
taxonRank	Taxonomic rank of the most specific name in the scientificName
scientificNameAuthorship	Authorship information for the scientificName
nomenclaturalCode	The nomenclatural code under which the scientificName is constructed
taxonomicStatus	Status of the use of the scientificName as a label for a taxon linked to http://www.tropicos.org/
modified	Date on which the resource was changed
language	The language of the resource.
rights	Information about who can access the resource or an indication of its security status
rightsHolder	The organization owning and managing rights over the resource
bibliographicCitation	Bibliography citing this dataset
datasetName	The name identifying the data set from which the record was derived
references	DOI of bibliography citing this dataset

## Additional information

### Environmental data coverage

**Description:** In Lagoa Seca, the dominant soil type is loamy sand, although five plots with sandy loam and a single plot with pure sand have been registered. In Calais, sandy loam dominates, but sandy clay loam was found in some plots (Table 5).

On average, the pH value in Lagoa Seca is lower than in Calais, which explains the higher availability of aluminum and the lower concentrations of phosphorus, potassium, calcium and magnesium. Furthermore, the cation exchange capacity of Lagoa Seca is higher than in Calais, whereas the saturation of bases is lower in Lagoa Seca than in Calais (Tables 6, 7).

### Description of the Darwin Core Archive containing dataset

Column labels and descriptions of further Darwin Core Archive files from both datasets are given at

Table 8 (distribution.txt)

Table 9 (habit.txt)

Table 10 (measurementorfactsoil.txt)

Table 11 (measurementorfactspeciescardinality.txt)

## Supplementary Material

Supplementary material 1 Lagoa Seca Data type: Darwin Core Archive
Brief description: 76 species occurrences within and environmental properties of the 15 plots of 10x10m from the Lagoa Seca study site.
File: oo_36864.zip Markus Gastauer, Werner Leyh, Angela S. Miazaki, João A.A. Meira-Neto

Supplementary material 2 Calais Data type: Darwin Core Archive
Brief description: 107 species occurrences within and environmental properties of the 15 plots of 10x10m from the Calais study site.
File: oo_36863.zip Markus Gastauer, Werner Leyh, Angela S. Miazaki, João A.A. Meira-Neto

## Figures and Tables

**Table 1.** Species cardinality in the Wilmanns cover-abundance scale (Reichelt and Wilmanns 1973) and its conversion to the Braun-Blanquet scale (Mueller-Dombois and Ellenberg 1974).

**Category**	**Species’ cardinality**	**Braun-Blanquet**
r	1 individual/shot	r
+	2-5 individuals/shots	+
1	6-50 individuals/shots, covering less than 5 %	1
2m	> 50 individuals/shots, covering less than 5 %	2
2a	Covering 5 to 15 %	2
2b	Covering 15 to 25 %	2
3	Covering 25 to 50 %	3
4	Covering 50 to 75 %	4
5	Covering 75 to 100 %	5

**Table 2.** Phytosociological table of Lagoa Seca (Latitude 20°25.96'S, Longitude 43°29.47'W, Altitude 1600 m ASL), Ouro Preto, Minas Gerais, Brazil, with species cardinality estimations according to Reichelt and Wilmanns (1973) (see Table 1 for details) surveyed on 12.10.2008 (plots 1-12) and 22.10.2008 (plots 13-15). The abbreviation ne is northeastern exposition, nw is northwestern exposition, s is southern exposition, se is southeastern exposition, sw is southwestern exposition and FO is frequency of occurrence, i.e., number of plots in which species occurred.

**Plot number**	1	2	3	4	5	6	7	8	9	10	11	12	13	14	15	
**Vegetation cover [%]**	27	70	65	70	70	58	60	65	80	70	60	55	35	65	30	
**Number of species**	29	24	23	16	15	25	21	19	24	23	21	28	31	19	31	
**Rocky outcrops [% of surface]**	60	2	0	0	0	25	15	8	0	5	3	17	55	10	80	
**Inclination**	25	30	10	10	5	10	3	15	50	8	35	20	3	35	20	
**Exposition**	nw	nw	sw	sw	se	se	s	ne	se	sw	se	se	se	sw	sw	
**Scientific name**	**Cover-abundance category according to Reichelt and Wilmanns (1973)**															**FO**
*Eryngium paniculatum* Cav. & Dombey ex F. Delaroche (Apiaceae)	+	.	.	.	.	.	.	.	+	+	+	+	+	1	1	8
*Hydrocotyle quinqueloba* Ruiz & Pav. (Araliaceae)	.	.	.	.	.	.	.	.	.	.	.	.	.	1	.	1
*Aristolochia* sp. (Aristolochiaceae)	2m	.	.	.	.	.	.	.	.	.	.	.	.	.	.	1
*Achyrocline satureioides* (Lam.) DC. (Asteraceae)	r	+	1	.	.	1	1	1	1	1	.	2m	1	1	2m	12
*Baccharis aphylla* (Vell.) DC. (Asteraceae)	.	.	.	.	.	.	.	.	.	1	.	.	+	.	1	3
*Baccharis platypoda* DC. (Asteraceae)	1	.	.	.	.	r	.	.	.	.	+	.	1	.	.	4
*Baccharis reticularia* DC. (Asteraceae)	.	.	.	.	.	+	.	.	.	.	.	.	1	.	.	2
*Baccharis serrulata* (Lam.) Pers. (Asteraceae)	+	.	.	.	.	.	1	.	.	.	.	+	2m	.	.	4
*Baccharis* sp. (Asteraceae)	.	.	.	.	.	.	.	.	.	+	.	.	.	.	.	1
*Eremanthus erythropappus* (DC.) MacLeish (Asteraceae)	+	.	.	.	.	.	r	.	.	.	.	1	.	+	r	5
*Eremanthus incanus* (Less.) Less. (Asteraceae)	.	.	.	.	.	.	.	.	.	.	.	.	+	.	+	2
*Koanophyllon adamantium* (Gardner) R.M. King & H. Rob. (Asteraceae)	.	.	.	.	.	.	.	.	.	.	.	.	.	1	.	1
*Mikania nummularia* DC. (Asteraceae)	.	2m	2a	1	1	1	1	1	2m	1	2m	1	.	1	1	13
*Mikania* sp. 2 (Asteraceae)	.	.	.	.	.	.	.	.	1	.	.	1	+	.	+	4
*Richterago amplexifolia* (Gardner) Kuntze (Asteraceae)	1	+	+	.	.	2m	.	.	.	+	1	2m	+	.	2m	9
*Senecio adamantinus* Bong. (Asteraceae)	.	r	+	.	+	.	.	.	1	.	.	.	.	.	.	4
*Stenocline* sp. (Asteraceae)	+	.	.	.	.	.	.	.	.	.	.	.	.	+	r	3
*Cryptanthus schwackeanus* Mez (Bromeliaceae)	+	.	.	.	.	r	.	.	.	.	.	.	.	.	2m	3
*Utricularia amethystina* Salzm. ex A. St.-Hil. & Girard (Lentibulariaceae)	.	.	.	r	.	.	.	.	.	.	.	.	.	.	.	1
*Genlisea repens* Benj. (Lentibulariaceae)	.	+	2m	2m	+	1	1	1	1	1	+	+	+	.	.	12
*Lobelia camporum* Pohl (Campanulaceae)	.	.	.	.	.	.	.	.	.	.	r	.	.	.	.	1
*Rhynchospora consanguinea* (Kunth) Boeckeler (Cyperaceae)	.	.	.	.	.	.	.	.	.	.	2m	.	.	.	.	1
*Fimbristylis* sp. (Cyperaceae)	.	.	.	.	.	.	.	.	.	2m	.	.	.	.	.	1
*Trilepis microstachya* (C.B. Clarke) H. Pfeiff. (Cyperaceae)	.	.	.	2m	2m	2m	.	.	.	2m	.	.	+	.	.	5
*Rhynchospora* sp.1 (Cyperaceae)	2m	2m	2m	2m	2m	2a	2a	2m	2m	2m	1	1	2m	2m	2m	15
*Rhynchospora* sp.2 (Cyperaceae)	.	.	2m	.	.	.	.	.	r	.	.	.	+	.	.	3
*Scleria hirtella* Sw. (Cyperaceae)	.	.	.	.	.	.	.	.	.	.	.	.	.	1	.	1
*Cyperaceae* sp. 1	.	.	.	.	.	1	1	.	.	.	.	.	.	.	2m	3
*Cyperaceae* sp. 2	.	2a	1	2a	2m	2m	2a	2a	2b	2m	2b	2a	.	.	.	11
*Cyperaceae* sp. 3	.	.	.	.	1	2m	2m	.	.	2a	2m	2m	+	.	.	7
*Doryopteris ornithopus* (Mett.) J. Sm. (Pteridaceae)	.	.	.	.	.	.	.	.	.	.	.	.	.	.	1	1
*Drosera montana* A. St.-Hil. (Droseraceae)	1	2m	2m	2m	1	2m	1	+	.	1	2m	1	.	.	.	11
*Neomarica glauca* (Seub. ex Klatt) Sprague (Iridaceae)	1	+	1	.	.	.	.	.	.	.	.	+	1	.	.	5
*Mesosphaerum homalophyllum* (Pohl ex Benth.) Kuntze (Lamiaceae)	1	.	.	.	.	+	.	+	1	.	+	1	1	1	1	9
*Hyptis monticola* Mart. ex Benth. (Lamiaceae)	r	1	1	.	1	+	1	1	.	1	1	1	1	.	1	12
*Byrsonima variabilis* A. Juss. (Malpighiaceae)	1	r	.	.	.	+	.	+	.	.	.	.	+	.	+	6
*Cambessedesia hilariana* (Kunth) DC. (Melastomataceae)	1	1	1	.	.	1	+	1	.	.	.	.	1	.	.	7
*Lavoisiera* sp. (Melastomataceae)	.	.	+	.	.	.	.	.	.	.	.	.	.	.	.	1
*Leandra australis* (Cham.) Cogn. (Melastomataceae)	r	.	.	.	.	.	.	.	.	.	.	.	+	.	+	3
*Microlicia crenulata* (DC.) Mart. (Melastomataceae)	1	2m	.	.	.	.	1	+	1	.	.	+	2m	.	.	7
*Microlicia* sp. 1 (Melastomataceae)	2m	1	.	.	.	.	1	.	.	.	2m	1	.	1	.	6
*Microlicia* sp. 2 (Melastomataceae)	.	.	.	.	.	.	.	.	1	.	.	+	.	.	.	2
*Tibouchina cardinalis* Cogn. (Melastomataceae)	.	.	.	.	.	1	.	.	.	.	.	.	.	.	.	1
*Ardisia* sp. (Primulaceae)	.	.	.	.	.	.	.	.	.	.	.	.	.	r	.	1
*Myrsine umbellata* Mart. (Primulaceae)	.	.	.	.	.	.	.	.	+	.	.	.	.	.	r	2
*Myrcia eriocalyx* DC. (Myrtaceae)	.	.	.	.	.	.	.	.	.	.	.	.	.	.	+	1
*Myrcia splendens* (Sw.) DC. (Myrtaceae)	.	.	.	.	.	.	.	.	.	.	.	.	.	.	+	1
*Myrcia subcordata* DC. (Myrtaceae)	r	.	.	.	.	.	.	.	.	.	.	.	.	.	.	1
*Epidendrum denticulatum* Barb. Rodr. (Orchidaceae)	.	.	.	.	.	.	.	.	r	.	.	.	.	.	.	1
*Habenaria rupicola* Barb. Rodr. (Orchidaceae)	.	r	r	.	.	1	.	.	.	.	.	r	1	.	.	5
*Habenaria* sp. (Orchidaceae)	.	.	.	.	.	.	.	.	.	.	.	.	.	.	r	1
*Coppensia blanchetii* (Rchb. f.) Campacci (Orchidaceae)	.	.	.	.	.	.	r	.	.	.	.	.	.	.	r	2
*Coppensia warmingii* (Rchb. f.) Campacci (Orchidaceae)	.	.	.	.	.	.	.	.	.	.	.	.	.	.	r	1
*Sophronitis* sp. (Orchidaceae)	.	.	.	.	.	.	.	.	.	.	.	.	.	.	r	1
*Aristida* sp. (Poaceae)	.	2m	2m	2a	1	.	.	1	2a	.	.	.	.	2m	.	7
*Ichnanthus bambusiflorus* (Trin.) Döll (Poaceae)	2m	.	.	.	.	.	.	.	.	.	.	.	+	.	2m	3
*Otachyrium versicolor* (Döll) Henrard (Poaceae)	.	1	2m	2a	2m	.	.	2m	1	1	1	.	.	.	.	8
*Panicum pseudisachne* Mez (Poaceae)	2m	2a	2a	2m	.	2a	2m	2b	2m	2m	2m	2a	2m	2a	2m	14
*Panicum wettsteinii* Hack. (Poaceae)	2m	2a	2a	.	2m	.	1	2m	2a	1	1	2m	1	2a	2m	13
*Paspalum coryphaeum* Trin. (Poaceae)	2a	2b	2a	1	2a	2m	2a	2a	2a	2a	2m	2a	2m	2a	1	15
*Paspalum multicaule* Poir. (Poaceae)	.	.	.	2m	2a	2m	.	.	2a	1	.	+	.	.	.	6
*Schizachyrium sanguineum* (Retz.) Alston (Poaceae)	2m	2a	2m	1	2m	2m	2m	2m	2m	2m	+	2m	2m	2m	2m	15
*Sporobolus metallicola* Longhi-Wagner & Boechat (Poaceae)	.	2m	2m	1	.	.	.	.	.	.	.	.	.	.	.	3
*Poaceae* sp. 1	.	.	.	.	.	.	.	.	.	.	.	.	.	.	2m	1
*Poaceae* sp. 2	.	.	.	.	.	.	.	.	.	.	.	1	+	.	.	2
*Polygala paniculata* L. (Polygalaceae)	2m	1	1	.	.	1	2m	2m	.	1	1	1	2m	.	.	10
Roupala montana var. paraensis (Huber) K.S. Edwards (Proteaceae)	.	.	.	.	.	.	.	.	+	.	.	.	.	.	.	1
*Selaginella* sp. (Selaginellaceae)	.	.	.	2m	.	.	.	.	.	.	.	.	.	.	.	1
*Smilax oblongifolia* Pohl ex Griseb. (Smilacaceae)	.	.	.	.	.	.	.	.	.	.	.	.	.	.	+	1
*Brunfelsia brasiliensis* (Spreng.) L.B. Sm. & Downs (Solanaceae)	.	.	.	.	.	.	.	.	r	.	.	.	1	+	.	3
*Solanum granuloso-leprosum* Dunal (Solanaceae)	.	.	.	.	.	.	.	+	+	1	.	.	.	1	.	4
*Vellozia compacta* Mart. ex Schult. f. (Velloziaceae)	1	.	.	.	.	+	.	.	.	.	.	r	1	.	.	4
*Stachytarpheta commutata* Schauer (Verbenaceae)	+	.	.	.	.	.	.	.	.	.	.	.	.	.	.	1
*Xyris plantaginea* Mart. (Xyridaceae)	1	2m	2m	2m	.	.	1	.	.	1	2m	1	.	.	.	8
*Xyris* sp. 1 (Xyridaceae)	2m	2m	2m	2m	2a	2a	2m	2a	2m	2a	2m	2m	1	2m	.	14
*Xyris* sp. 2 (Xyridaceae)	.	.	.	.	.	.	.	.	.	.	.	1	+	1	.	3

**Table 3.** Phytosociological table of Calais (Latitude 20°24.61'S, Longitude 43°30.13'W, Altitude 1270 m ASL), Ouro Preto, Minas Gerais, Brazil, with species cover-abundance estimations according to Reichelt and Wilmanns (1973) (see Table 1 for details) surveyed on 09.02.2009 (plots 1-3) and 10.02.2009 (plots 4-15). The abbreviation e is eastern exposition, n is northern exposition, ne is northeastern exposition, nw is northeastern exposition, se is southeastern exposition, FO is frequency of occurrence, i.e., number of plots in which species occurred.

**Plot number**	1	2	3	4	5	6	7	8	9	10	11	12	13	14	15	
**Vegetation cover [%]**	97	85	75	65	75	90	75	75	70	89	60	70	80	75	75	
**Number of species**	45	35	50	39	23	30	34	28	38	29	30	24	27	31	32	
**Rocky outcrops [% of surface]**	10	5	25	60	60	0	0	3	5	0	15	25	25	40	35	
**Inclination**	3	5	20	25	15	0	10	15	20	5	20	20	10	20	15	
**Exposition**	e	se	se	se	se	-	-	ne	ne	-	n	nw	nw	nw	ne	
**Scientific name**	**Cover-abundance category according to Reichelt and Wilmanns (1973)​**															**FO**
*Acanthospermum australe* (Loefl.) Kuntze (Asteraceae)	.	.	r	.	.	.	.	.	1	1	.	.	.	.	+	4
*Achyrocline satureioides* (Lam.) DC. (Asteraceae)	2b	2a	1	.	.	1	+	.	.	.	+	1	1	+	.	9
*Aeschynomene elegans* Schltdl. & Cham. (Fabaceae)	1	1	.	.	.	+	+	.	.	.	.	.	.	.	.	4
*Amaranthaceae* sp. (Amaranthaceae)	.	.	.	.	.	.	.	.	r	.	.	.	.	.	.	1
*Andropogon leucostachyus* Kunth (Poaceae)	2m	1	2a	1	1	2m	2m	2a	1	.	2b	2m	2a	2a	1	14
Anemia ferruginea var. ahenobarba (Christ.) Mickel (Anemiaceae)	2m	2m	1	.	.	.	.	+	1	.	1	.	1	1	r	9
*Apochloa poliophylla* (Renvoize & Zuloaga) Zuloaga & Morrone (Poaceae)	.	.	.	.	.	.	.	.	1	.	.	.	.	.	.	1
*Axonopus siccus* (Nees) Kuhlm. (Poaceae)	1	2m	2m	1	.	2m	2a	2m	.	.	2m	2m	2m	2m	2m	12
*Baccharis dracunculifolia* DC. (Asteraceae)	.	+	.	.	.	.	.	.	.	.	.	.	.	.	.	1
*Baccharis serrulata* (Lam.) Pers. (Asteraceae)	.	1	1	.	1	.	2a	1	+	.	+	1	+	1	1	11
*Baccharis sessiliflora* Vahl (Asteraceae)	r	.	1	+	.	r	r	.	+	.	.	.	+	.	.	7
*Baccharis* sp. (Asteraceae)	2a	1	1	1	.	1	1	1	1	.	1	1	.	1	1	12
*Banisteriopsis campestris* (A. Juss.) Little (Malpighiaceae)	.	.	.	.	.	.	.	r	.	.	.	.	.	+	r	3
*Blechnum tabulare* (Thunb.) Kuhn (Blechnaceae)	.	.	r	r	.	.	.	.	.	.	.	.	.	.	.	2
*Byrsonima variabilis* A. Juss. (Malpighiaceae)	r	.	.	.	.	.	.	.	.	.	.	.	.	.	.	1
*Cambessedesia hilariana* (Kunth) DC. (Melastomataceae)	1	.	.	.	.	.	.	r	.	.	.	.	.	.	.	2
*Casearia sylvestris* Sw. (Salicaceae)	.	.	.	r	.	.	.	.	.	.	.	.	.	.	.	1
*Chamaecrista flexuosa* (L.) Greene (Fabaceae)	.	.	.	.	.	+	+	.	1	r	.	.	.	.	+	5
*Chamaecrista rotundifolia* (Pers.) Greene (Fabaceae)	r	1	1	.	.	.	.	.	.	.	.	.	.	.	r	4
*Chaptalia nutans* (L.) Pol. (Asteraceae)	.	.	.	.	.	.	.	.	.	+	.	.	.	.	.	1
*Chloris* sp. (Poaceae)	2a	2m	2m	.	.	2m	2m	2m	2m	.	1	+	2m	2m	1	12
*Crotalaria* sp. (Fabaceae)	.	.	+	.	.	.	.	.	.	.	.	.	.	.	.	1
*Cuphea carthagenensis* (Jacq.) J.F. Macbr. (Lythraceae)	.	.	+	.	.	.	.	.	.	.	.	.	.	.	.	1
*Cuphea* sp. (Lythraceae)	r	.	.	r	.	.	.	.	.	.	.	.	.	.	.	2
*Cyperaceae* sp. 1	.	.	.	2b	2m	.	.	.	.	.	.	.	.	.	.	2
*Cyperaceae* sp. 3	.	.	.	.	.	2m	1	.	.	.	+	.	1	1	.	5
*Cyperaceae* sp. 4	.	.	.	.	.	.	.	.	.	2m	.	.	.	.	.	1
*Cyrtocymura scorpioides* (Lam.) H. Rob. (Asteraceae)	r	1	1	.	2m	1	2a	1	+	.	1	+	.	+	.	11
*Dalbergia brasiliensis* Vogel (Fabaceae)	+	+	2a	+	.	r	.	r	.	.	r	r	.	.	r	9
*Desmodium adscendens* (Sw.) DC. (Fabaceae)	.	1	+	.	.	.	.	.	.	1	.	.	.	.	.	3
*Desmodium barbatum* (L.) Benth. (Fabaceae)	.	.	.	.	.	.	.	.	.	r	.	.	.	.	.	1
*Dichorisandra thyrsiflora* J.C. Mikan (Commelinaceae)	.	.	.	r	.	.	.	.	.	.	.	.	.	.	.	1
*Dicranopteris flexuosa* (Schrad.) Underw. (Gleicheniaceae)	.	.	r	.	.	.	.	.	+	.	.	.	+	.	.	3
*Dictyoloma vandellianum* A.H.L. Juss. (Rutaceae)	.	.	r	.	.	.	.	.	.	.	.	.	.	.	.	1
*Diodia teres* Walter (Rubiaceae)	.	.	.	1	+	.	.	.	.	.	.	.	.	.	.	2
*Dioscorea* sp. (Dioscoreaceae)	.	.	r	.	r	.	.	.	.	.	.	.	.	.	.	2
*Diplusodon buxifolius* Cham. & Schltdl. (Lythraceae)	.	.	+	.	.	.	.	1	1	.	.	.	r	.	.	4
*Eragrostis maypurensis* (Kunth) Steud. (Poaceae)	1	.	.	.	.	1	.	.	.	.	.	.	.	.	.	2
*Eremanthus crotonoides* (DC.) Sch. Bip. (Asteraceae)	.	1	.	.	.	.	.	.	.	.	.	.	.	.	.	1
*Eremanthus erythropappus* (DC.) MacLeish (Asteraceae)	.	.	r	.	.	.	r	.	.	.	r	.	.	.	.	3
*Eremanthus incanus* (Less.) Less. (Asteraceae)	+	.	2a	.	.	r	+	.	.	.	+	.	2a	.	r	7
*Eupatorium* sp. (Asteraceae)	r	+	+	r	1	.	.	.	.	.	r	.	.	+	1	8
*Fabaceae* sp.	.	.	.	.	2a	.	.	.	.	.	.	.	.	.	.	1
*Guatteria villosissima* A. St.-Hil. (Annonaceae)	.	.	.	r	.	.	.	.	.	.	.	r	1	.	.	3
*Heteropterys* sp. (Malpighiaceae)	.	+	r	.	.	+	+	.	r	r	+	+	r	.	.	9
*Hypoxis decumbens* L. (Hypoxidaceae)	.	.	.	.	.	.	.	.	.	r	.	.	.	.	.	1
*Ichnanthus bambusiflorus* (Trin.) Döll (Poaceae)	.	.	.	.	.	.	.	.	.	.	.	.	1	2m	.	2
*Inga sessilis* (Vell.) Mart. (Fabaceae)	.	.	.	r	.	.	.	.	.	.	.	.	.	.	.	1
*Lantana camara* L. (Verbenaceae)	.	.	.	1	1	.	.	.	.	1	.	.	.	.	.	3
*Lantana fucata* Lindl. (Verbenaceae)	.	.	+	+	.	.	.	.	r	+	r	.	.	.	r	6
*Lippia hermannioides* Cham. (Verbenaceae)	.	1	1	r	.	.	.	+	.	+	1	.	1	1	.	8
*Matayba marginata* Radlk. (Sapindaceae)	+	.	2a	1	1	+	+	+	+	.	r	r	r	+	.	12
*Melinis minutiflora* P. Beauv. (Poaceae)	4	4	3	3	.	3	3	3	3	3	.	4	3	3	3	13
*Mesosphaerum homalophyllum* (Pohl ex Benth.) Kuntze (Lamiaceae)	1	.	+	.	.	.	r	+	+	.	r	+	.	.	1	8
*Miconia pepericarpa* Mart. ex DC. (Melastomataceae)	.	+	.	.	.	+	+	r	1	+	+	.	.	r	+	9
*Miconia* sp. 1 (Melastomataceae)	1	1	+	.	.	.	r	.	+	.	.	.	.	.	.	5
*Miconia* sp. 2 (Melastomataceae)	+	+	+	.	.	+	r	1	.	.	+	r	2a	+	+	11
*Microlicia* sp. 3 (Melastomataceae)	1	+	.	.	.	1	1	1	.	.	2b	.	.	.	.	6
*Mikania nummularia* DC. (Asteraceae)	1	r	+	+	.	.	+	.	r	.	r	1	.	+	.	9
*Myrcia amazonica* DC. (Myrtaceae)	.	.	r	.	.	.	.	.	.	.	.	.	.	.	.	1
*Myrcia splendens* (Sw.) DC. (Myrtaceae)	.	.	r	.	.	.	.	.	.	.	.	.	.	.	.	1
*Oxypetalum appendiculatum* Mart. (Apocynaceae)	.	+	.	.	r	.	.	.	+	.	.	.	.	.	.	3
*Panicum pseudisachne* Mez (Poaceae)	.	.	1	.	.	1	1	.	.	.	.	.	2a	2m	1	6
*Panicum wettsteinii* Hack. (Poaceae)	1	1	2m	.	2a	.	2m	1	2m	2m	.	.	2m	2m	1	11
*Paspalum hyalinum* Nees ex Trin. (Poaceae)	1	2m	.	1	.	.	.	.	.	.	.	.	.	.	.	3
*Paspalum notatum* Alain ex Flüggé (Poaceae)	.	.	.	.	.	.	.	.	.	2a	.	.	.	.	.	1
*Paspalum plicatulum* Michx. (Poaceae)	.	.	.	.	+	.	2m	2m	1	+	2m	.	.	1	.	7
*Pecluma pectinata* (L.) M.G. Price (Polypodiaceae)	.	.	.	1	.	.	.	.	.	.	.	.	.	.	.	1
*Pennisetum setosum* (Sw.) Rich. (Poaceae)	1	1	.	.	.	.	.	.	.	.	.	.	.	.	.	2
*Periandra mediterranea* (Vell.) Taub. (Fabaceae)	1	.	.	.	2m	r	1	1	+	1	.	.	r	r	.	9
*Poa annua* L. (Poaceae)	.	.	.	1	.	.	.	.	2a	2m	.	.	.	.	.	3
*Poaceae* sp. 2	1	.	.	2m	.	.	.	1	.	.	.	.	.	.	.	3
*Poaceae* sp. 3	2m	.	.	.	.	1	.	.	.	.	1	.	.	1	1	5
*Polygala paniculata* L. (Polygalaceae)	1	.	2m	2m	.	.	1	2m	2m	1	.	.	.	.	1	8
*Polygala violacea* Aubl. (Polygalaceae)	+	.	.	.	.	.	.	.	r	+	.	.	.	.	.	3
*Pteridium arachnoideum* (Kaulf.) Maxon (Dennstaedtiaceae)	r	.	.	.	.	.	+	.	.	.	.	+	.	.	.	3
*Pterocaulon lanatum* Kuntze (Asteraceae)	.	+	.	.	.	.	.	.	.	1	.	.	.	.	.	2
*Rhynchospora corymbosa* (L.) Britton (Cyperaceae)	.	.	1	1	.	.	.	.	.	.	.	+	.	.	.	3
*Rhynchospora* sp. 3 (Cyperaceae)	.	.	.	.	.	.	.	.	.	1	.	.	.	.	.	1
*Rhynchospora tenuis* Willd. ex Link (Cyperaceae)	.	.	1	.	.	.	.	.	.	.	.	.	.	.	.	1
*Rubus brasiliensis* Mart. (Rosaceae)	.	.	.	.	.	.	.	.	.	.	.	.	r	.	.	1
*Ruellia macrantha* Lindau (Acanthaceae)	.	.	.	r	r	.	.	.	.	.	.	.	.	.	.	2
*Sacoila lanceolata* (Aubl.) Garay (Orchidaceae)	.	.	.	r	.	.	.	.	.	.	.	.	.	.	.	1
*Schizachyrium sanguineum* (Retz.) Alston (Poaceae)	2m	.	.	.	.	2a	.	.	2m	.	.	.	.	.	.	3
*Schwenckia americana* D. Royen ex L. (Solanaceae)	.	.	1	.	.	.	.	.	.	.	.	.	.	.	2m	2
*Scleria hirtella* Sw. (Cyperaceae)	2m	2m	.	1	.	1	2m	.	1	.	.	.	2a	.	.	7
*Scleria* sp. (Cyperaceae)	.	.	.	.	.	.	.	2a	.	.	.	.	.	.	.	1
*Senecio adamantinus* Bong. (Asteraceae)	.	.	.	1	1	.	.	.	.	+	.	r	.	.	.	4
*Senna reniformis* (G. Don) H.S. Irwin & Barneby (Fabaceae)	.	.	.	.	.	.	.	.	.	.	.	r	.	.	.	1
*Sida linifolia* Cav. (Malvaceae)	+	+	+	+	+	.	.	.	.	r	.	.	.	.	.	6
*Sisyrinchium vaginatum* Spreng. (Iridaceae)	.	.	+	.	.	.	.	.	.	.	.	.	.	.	.	1
*Solanum americanum* Mill. (Solanaceae)	.	.	.	r	.	.	.	.	.	.	.	.	.	.	.	1
*Solanum granuloso-leprosum* Dunal (Solanaceae)	.	.	.	2a	2m	.	.	.	2m	2b	.	r	.	r	+	7
*Spermacoce verticillata* L. (Rubiaceae)	1	+	1	1	1	+	.	.	+	1	+	.	.	+	+	11
*Sporobolus metallicola* Longhi-Wagner & Boechat (Poaceae)	.	.	.	.	.	.	.	.	.	.	.	.	.	+	.	1
*Stevia clausseni* Sch. Bip. ex Baker (Asteraceae)	.	.	.	.	.	.	.	.	2a	.	.	.	.	.	.	1
*Stylosanthes viscosa* (L.) Sw. (Fabaceae)	1	1	r	.	.	.	.	.	r	.	.	r	.	2a	1	7
*Tibouchina heteromalla* (D. Don) Cogn. (Melastomataceae)	.	.	+	.	.	.	+	r	.	.	r	.	.	.	r	5
*Tradescantia ambigua* Mart. (Commelinaceae)	.	.	+	+	.	1	.	.	.	.	.	.	.	.	.	3
*Trichogonia* sp. (Asteraceae)	r	.	+	1	.	.	+	1	+	.	+	1	.	+	r	10
*Trilepis microstachya* (C.B. Clarke) H. Pfeiff. (Cyperaceae)	.	.	.	1	.	.	.	.	.	1	.	.	.	.	2a	3
*Urochloa decumbens* (Stapf) R.D. Webster (Poaceae)	1	.	.	.	2a	.	.	.	.	.	.	.	1	.	.	3
*Varronia curassavica* Jacq. (Boraginaceae)	+	.	+	r	1	.	r	r	.	+	r	.	+	+	+	11
*Vernonia* sp. 1 (Asteraceae)	1	+	.	.	.	.	.	.	+	.	.	.	.	.	.	3
*Vernonia* sp. 2 (Asteraceae)	.	.	.	.	.	2a	.	.	.	.	+	.	.	.	.	2
*Wissadula* sp. (Malvaceae)	.	.	r	.	.	.	.	.	+	.	r	.	+	r	+	6
*Zornia reticulata* Sm. (Fabaceae)	+	+	r	r	.	r	.	.	.	+	.	.	.	.	.	6

**Table 4.** Description of the files from the Darwin Core Archive **dwca-camporupestre-15plot-survey-sampling-itacolomi-lagoa076-checklist.zip** (Suppl. material 1) and d**wca-camporupestre-15plot-survey-sampling-itacolomi-calais107-checklist.zip** (Suppl. material 2)

**file**	**Description**
taxon.txt	core taxon file, contains a list of taxa occurring in this dataset
meta.xml	(DwC-) archive descriptor
measurementorfactsoil.txt	extension file: measurements or facts, description of the physical and chemical soil properties in plots where taxa were registered (due to technical issues, extension files measurementorfactsoil.txt and measurementorfactspeciescardinality.txt were merged to the single extension file measurementorfact.txt)
measurementorfactspeciescardinality.txt	extensionfile: describes the cardinaltiy of species occurrences within the 15 plots of 10 x 10 m within each study site (due to technical issues, extension files measurementorfactsoil.txt and measurementorfactspeciescardinality.txt were merged to the single extension file measurementorfact.txt)
description.txt	extension file: habitat, contains habitat type in which the taxon was registered
eml.xml	meta data document
distribution.txt	extension file: describes the taxa occurrence within the 15 plots of 10 x 10 m within each data set

**Table 5.** Physical soil properties examined in each of the 15 plots of 10 x 10 m in Lagoa Seca (Latitude 20°26'S, Longitude 43°29'W, Altitude 1600 m ASL) and Calais (Latitude 20°25'S, Longitude 43°30'W, Altitude ASL 1270 m), Ouro Preto, Minas Gerais, Brazil. Plot denomination is consistent with that from Table 2 and Table 3​.

**Plot**	**Coarse sand [%]**	**Fine sand [%]**	**Silt [%]**	**Clay [%]**	**Soil type**
						**Lagoa Seca**					
1	32	44	15	9	Sandy loam
2	32	45	14	9	Sandy loam
3	29	45	18	8	Sandy loam
4	28	51	13	8	Sandy loam
5	34	56	6	4	Sand
6	33	49	9	9	Loamy sand
7	34	47	13	6	Loamy sand
8	33	45	14	8	Sandy loam
9	27	52	12	9	Loamy sand
10	36	45	9	10	Loamy sand
11	27	54	14	5	Loamy sand
12	32	45	12	11	Sandy loam
13	29	46	16	9	Sandy loam
14	30	45	11	14	Sandy loam
15	30	43	16	11	Sandy loam
**Calais**					
1	39	34	15	12	Sandy loam
2	33	37	18	12	Sandy loam
3	36	36	16	12	Sandy loam
4	35	35	18	12	Sandy loam
5	35	32	19	14	Sandy loam
6	32	42	18	8	Sandy loam
7	43	34	16	7	Sandy loam
8	44	34	15	7	Sandy loam
9	39	35	18	8	Sandy loam
10	35	34	24	7	Sandy clay loam
11	38	37	18	7	Sandy loam
12	32	33	19	16	Sandy loam
13	34	34	26	6	Sandy clay loam
14	38	42	16	4	Sandy loam
15	39	38	20	3	Sandy clay loam

**Table 6.** Measured pH, nutrient and aluminum availability as well as potential acidity in each of the 15 plots of 10 x 10 m in Lagoa Seca (Latitude 20°26'S, Longitude 43°29'W, Altitude 1600 m ASL) and Calais (Latitude 20°25'S, Longitude 43°30'W, Altitude ASL 1270 m), Ouro Preto, Minas Gerais, Brazil. Plot denomination is consistent with that from Table 2 and Table 3.

**Plot**	**pH**	**Availability of**					**Potential acidity [cmol_c_/ dm^3^]**
		**Phosphorus [mg/ dm^3^]**	**Potassium [mg/ dm^3^]**	**Ca^2+ ^[cmol_c_/ dm^3^]**	**Mg^2+ ^[cmol_c_/ dm^3^]**	Al^3+^* [cmol_c _/dm^3^]	
**Lagoa Seca**							
**1**	4.47	0.9	11	0.1	0.05	1.93	6.2
**2**	4.35	2.3	19	0.21	0.06	2.03	8.7
**3**	4.39	2	16	0.2	0.07	1.07	6.5
**4**	4.41	2.2	18	0.38	0.1	1.61	9.4
**5**	5.12	1.6	12	0.27	0.08	1.82	5.5
**6**	4.74	1.2	12	0.16	0.04	2.35	7.1
**7**	4.5	1	13	0.14	0.04	1.28	4.6
**8**	4.67	1.1	17	0.31	0.08	2.25	8.7
**9**	4.7	2.2	16	0.26	0.08	2.03	9.9
**10**	4.81	1.6	16	0.22	0.06	2.14	8.1
**11**	4.87	1.9	14	0.21	0.06	1.18	5.8
**12**	4.81	0.9	9	0.15	0.04	1.82	7.4
**13**	4.4	1.3	11	0.18	0.06	2.03	6.9
**14**	4.52	1.6	20	0.14	0.05	3.1	8.3
**15**	4.51	1.2	13	0.16	0.05	3.1	8.8
**Calais**							
**1**	5.12	1.4	30	0.36	0.09	1.18	4.6
**2**	5.05	1.7	33	0.33	0.12	1.18	4.9
**3**	5.35	2.2	34	0.4	0.14	1.07	5.3
**4**	5.09	2.2	41	1.01	0.17	0.96	6.4
**5**	4.8	2.2	22	0.53	0.08	2.25	8.5
**6**	5.27	1.7	39	0.17	0.06	1.61	3.4
**7**	5.17	1.6	30	0.3	0.08	1.07	5.3
**8**	5.02	1.4	24	0.14	0.06	1.18	3.7
**9**	4.86	1.1	11	0.06	0.03	1.07	3.7
**10**	6.05	2.2	24	1.19	0.56	0	1.9
**11**	5.2	1.5	21	0.17	0.05	0.96	3.7
**12**	4.76	1.6	26	0.2	0.06	1.61	6.5
**13**	5.64	1.6	24	0.33	0.09	0.43	2.1
**14**	5.52	1.3	16	0.16	0.06	0.54	2.3
**15**	5.69	1.1	9	0.27	0.11	0.11	1.1

**Table 7.** Amount of interchangeable bases, effective cation exchance capacity (CEC), saturation of bases and aluminum as well as remaining phosphorus in each of the 15 plots of 10 x 10 m in Lagoa Seca (Latitude 20°26'S, Longitude 43°29'W, Altitude 1600 m ASL) and Calais (Latitude 20°25'S, Longitude 43°30'W, Altitude ASL 1270 m), Ouro Preto, Minas Gerais, Brazil. Plot denomination is consistent with that from Table 2 and Table 3.

**Plot**	**Interchangeable bases [cmol_c_/** **dm^3^]**	**Effective CEC [cmol_c_/ dm^3^]**	**CEC (pH 7,0) [cmol_c_/ dm^3^]**	**Saturation of bases [%]**	**Saturation of Al [%]**	**Remaining phosphorus [mg/L]**
**Lagoa Seca**						
**1**	0.18	2.11	6.38	2.8	91.5	37.3
**2**	0.32	2.35	9.02	3.5	86.4	37.6
**3**	0.31	1.38	6.81	4.6	77.5	36
**4**	0.53	2.14	9.93	5.3	75.2	40.3
**5**	0.38	2.2	5.88	6.5	82.7	45.5
**6**	0.23	2.58	7.33	3.1	91.1	34.9
**7**	0.21	1.49	4.81	4.4	85.9	44.2
**8**	0.43	2.68	9.13	4.7	84	37.6
**9**	0.38	2.41	10.28	3.7	84.2	35.5
**10**	0.32	2.46	8.42	3.8	87	32.2
**11**	0.31	1.49	6.11	5.1	79.2	47.4
**12**	0.21	2.03	7.61	2.8	89.7	33.3
**13**	0.27	2.3	7.17	3.8	88.3	38.4
**14**	0.24	3.34	8.54	2.8	92.8	28.6
**15**	0.24	3.34	9.04	2.7	92.8	30.7
**Calais**						
**1**	0.53	1.71	5.13	10.3	69	37.8
**2**	0.53	1.71	5.43	9.8	69	38
**3**	0.63	1.7	5.93	10.6	62.9	38.3
**4**	1.28	2.24	7.68	16.7	42.9	36.5
**5**	0.67	2.92	9.17	7.3	77.1	31.7
**6**	0.33	1.94	3.73	8.8	83	39
**7**	0.46	1.53	5.76	8	69.9	42.1
**8**	0.26	1.44	3.96	6.6	81.9	42.7
**9**	0.12	1.19	3.82	3.1	89.9	43.4
**10**	1.81	1.92	3.71	48.8	5.7	46.9
**11**	0.27	1.23	3.97	6.8	78	46
**12**	0.33	1.94	6.83	4.8	83	28.4
**13**	0.48	0.91	2.58	18.6	47.3	47.4
**14**	0.26	0.8	2.56	10.2	67.5	52.6
**15**	0.4	0.51	1.5	26.7	21.6	55.5

**Table 8.** Column labels and descriptions of distribution.txt from Darwin Core Archives **dwca-camporupestre-15plot-survey-sampling-itacolomi-lagoa076-checklist.zip** (Suppl. material 1) and **dwca-camporupestre-15plot-survey-sampling-itacolomi-calais107-checklist.zip** (Suppl. material 2) containing the ​taxa occurrences within 15 plots of 10 x 10 in both study sites

**Column label**	**Column description**
Id	Taxon identifier
locationID	Identifier for the set of location information, here composed of plot name, decimal latitude and longitude
locality	Specific description of the locality
countryCode	Standard code for the country in which the location occurs
occurrenceStatus	Statement about the presence or absence of the taxon at the location
establishmentMeans	Process by which the taxon became established at the location
eventDate	Date-time at which the taxon was registered at the location
source	Related resource from which the described resource is derived, here the DOI of bibliography citing this dataset
occurrenceRemarks	Further comments or notes about the occurrence of the taxon at the location

**Table 9.** Column labels and descriptions of description.txt from Darwin Core Archives **dwca-camporupestre-15plot-survey-sampling-itacolomi-lagoa076-checklist.zip** (Suppl. material 1) and **dwca-camporupestre-15plot-survey-sampling-itacolomi-calais107-checklist.zip** (Suppl. material 2​) containing a description of habitat type in which taxa were registered.

Column label	Column description
Id	Taxon identifier
description	Habitat type, i.e., campo rupestre vegetation, at location where taxon was registered
type	The kind of description
language	The language of the ressource

**Table 10.** Column labels and descriptions of measurementorfactsoil.txt from Darwin Core Archives **dwca-camporupestre-15plot-survey-sampling-itacolomi-lagoa076-checklist.zip** (Suppl. material 1) and **dwca-camporupestre-15plot-survey-sampling-itacolomi-calais107-checklist.zip** (Suppl. material 2​) containing analysis of soil samples related to taxa occurrences.

Column label	Column description
Id	Taxon identifier
measurementType	Description of the measurement, these are clay, silt, fine and course sand content; pH; phosphorus (P), potassium (K), aluminium (Al), calcium (Ca) and magnesium (Mg) availability; potential acidity; saturation of bases and of aluminium; effective cation exchange capacity; cation exchange capacity at pH 7.0; interchangeable bases; remaining phosphorus; percentage of rocky outcrops; plot inclination
measurementValue	Value of the measurement
measurementUnit	Units associated with the measurementValue
measurementDeterminedDate	Date on which the measurement was carried out
measurementMethod	Description of the method used to determine soil properties
measurementRemarks	Comments or notes accompanying the measurement

**Table 11.** Column labels and descriptions of measurementorfactspeciescardinality.txt from Darwin Core Archives**dwca-camporupestre-15plot-survey-sampling-itacolomi-lagoa076-checklist.zip** (Suppl. material 1) and **dwca-camporupestre-15plot-survey-sampling-itacolomi-calais107-checklist.zip** (Suppl. material 2​) containing analysis of soil samples related to taxa occurrences.

**Column label**	**Column description**
Id	Taxon identifier
measurementType	Description of the measurement, this is estimation of species cardinality of taxa within 10 x 10 m plots according to the Wilmanns cover-abundance scale (Reichelt and Wilmanns 1973)
measurementValue	Value of the measurement
measurementUnit	Category of species cardinality
measurementDeterminedDate	Date on which the measurement was carried out
measurementMethod	Description of the method used to determine soil properties
measurementRemarks	Comments or notes accompanying the measurement
